# Father for the first time - development and validation of a questionnaire to assess fathers’ experiences of first childbirth (FTFQ)

**DOI:** 10.1186/1471-2393-12-35

**Published:** 2012-05-17

**Authors:** Åsa Premberg, Charles Taft, Anna-Lena Hellström, Marie Berg

**Affiliations:** 1 Institute of Health and Care Sciences, Sahlgrenska Academy at Gothenburg University, Box 457, SE-40530, Gothenburg, Sweden

**Keywords:** Fathers, Childbirth, Questionnaire, Validity, Reliability

## Abstract

**Background:**

A father’s experience of the birth of his first child is important not only for his birth-giving partner but also for the father himself, his relationship with the mother and the newborn. No validated questionnaire assessing first-time fathers' experiences during childbirth is currently available. Hence, the aim of this study was to develop and validate an instrument to assess first-time fathers’ experiences of childbirth.

**Method:**

Domains and items were initially derived from interviews with first-time fathers, and supplemented by a literature search and a focus group interview with midwives. The comprehensibility, comprehension and relevance of the items were evaluated by four paternity research experts and a preliminary questionnaire was pilot tested in eight first-time fathers. A revised questionnaire was completed by 200 first-time fathers (response rate = 81%) Exploratory factor analysis using principal component analysis with varimax rotation was performed and multitrait scaling analysis was used to test scaling assumptions. External validity was assessed by means of known-groups analysis.

**Results:**

Factor analysis yielded four factors comprising 22 items and accounting 48% of the variance. The domains found were *Worry, Information, Emotional support and Acceptance.* Multitrait analysis confirmed the convergent and discriminant validity of the domains; however, Cronbach’s alpha did not meet conventional reliability standards in two domains. The questionnaire was sensitive to differences between groups of fathers hypothesized to differ on important socio demographic or clinical variables.

**Conclusions:**

The questionnaire adequately measures important dimensions of first-time fathers’ childbirth experience and may be used to assess aspects of fathers’ experiences during childbirth. To obtain the FTFQ and permission for its use, please contact the corresponding author.

## Background

Becoming a father for the first time is a central episode in life [1-3]. Since fathers entered the delivery room some decades ago, their role has mainly been to give emotional support to the birth giving woman [4,5]. Nonetheless, attending childbirth also has benefits for the father. For example, it has been shown to facilitate the transition to fatherhood [6,7], solidify the father’s relationship with his birth giving partner [8-10], and promote early attachment between father and infant and their bonding [1,11].

On the other hand, fathers sometimes find childbirth more emotional and demanding than expected [12] and their need of personal support is well recognized [13,14]. In interviews with first-time fathers, we found that fathers oftentimes feel compelled to hide their feelings of insecurity, nervousness, irritation and frustration behind a confident, calm façade when supporting their partner during childbirth [15-17]. Some fathers also feel that they are unimportant during childbirth and feel ignored by midwives and other staff members. Fathers also frequently consider that the information they receive from midwives is conciliatory or erroneous, which has also been found in earlier research on fathers-to-be [18].

Little attention has focused on how such experiences impact on the fathers’ supportive role during labour and childbirth or subsequently on their role as fathers after childbirth. However, two recent studies indicate that fathers’ negative experiences during childbirth may be associated with depressive symptomatology after childbirth [19,20]. There is therefore a need to further investigate fathers’ childbirth experiences both to address the fathers own needs of support during childbirth and to identify fathers whose needs of support have not been met.

Questionnaires for assessing fathers’ experiences of the pregnancy period and childbirth have been developed [21-26]; however, there is only one validated instrument for assessing fathers’ experiences during labour and birth [6]. Given that first-time fathers are presumably particularly vulnerable during childbirth, an instrument designed specifically for assessing their experiences seems called for. The aim of this study was thus to develop and validate an instrument to assess first-time fathers' experiences of childbirth (FTFQ).

## Methods

### Design and ethical considerations

In this study, a methodological and developmental design was used to determine the construct validity and the internal consistency reliability of a questionnaire to assess first-time fathers’ experiences of childbirth. Permission to undertake the study was obtained from the responsible managers at the hospitals involved. The study was approved by the Regional Ethical Review Board in Gothenburg and was conducted in accordance with ethical principles of the Declaration of Helsinki [27].

### Instrument development

Procedures for developing the instrument encompassed three stages, 1. identification of important domains of the first-time father's experiences of childbirth and generation of items to represent the domains, 2. evaluation of comprehensiveness, comprehension and relevance of the items and 3. validation of the scales.

#### Identification of core domains

Five domains were identified from our previous interview studies with first-time fathers: ’expectations and wishes’, ‘information’, ‘support to the woman’, ‘emotional support’ and ‘comfort’ [15-17]. A literature search in Pub Med, Cinahl and Scopus using the keywords father, childbirth and questionnaire were conducted to identify other possible domains. A focus group interview was conducted with eight experienced female midwives to gain information about midwives perceptions of the fathers’ presence in labour and birth. The midwives were encouraged to freely discuss their impressions about how fathers experienced birth and fathers’ needs for support.

#### Item generation

Statements illustrating the five salient domains were extracted verbatim from our interviews with first-time fathers by two of the authors (ÅP and MB) [15,16]. Additional items were gleaned from our focus group interviews with midwives and from discussions with a panel of four experts in paternity research from different academic disciplines (midwifery, sociology and psychology).

#### Item evaluation

The resulting item pool was first scrutinized by the expert panel for comprehensiveness, relevance and comprehensibility. After modifications based on the panel’s recommendations, a preliminary version of the questionnaire was drafted. A 4-point Likert response scale was used (strongly agree (1) agree (2) slightly agree (3) disagree (4)).

The questionnaire was mailed to a pilot group of eight first-time fathers. They were asked to fill in the questionnaire and to rate the relevance and comprehensibility of each of the items on a 4-point scale. Items with low relevance ratings were omitted and those with low comprehensibility ratings were reworded. Thereafter the expert panel re-evaluated the questionnaire.

### Instrument validation

#### Setting and study sample

The study population comprised first-time fathers whose partner had given birth at one of four delivery wards at two major hospitals (one urban and one provincial) in southwestern Sweden between November 26 and December 24, 2009 (urban hospital) or between November 26 and January 9, 2010 (provincial hospital). As only mothers' postal addresses are documented in birth records in Sweden, first-time fathers were contacted via their birth giving partners. In February 2010, questionnaires were sent to 306 mothers who met the following inclusion criteria: first-time mothers with a vaginal birth (normal or instrumental) or an acute caesarean, who had reported a man as their closest relative and whose infant had an Apgar score over five assessed after five minutes. The mothers were mailed a letter requesting them to give the questionnaire to the father of the newborn. The fathers were requested to complete and return the paper questionnaire or to complete an electronic version via the internet. Only first-time fathers who were Swedish speaking and who had attended childbirth were included.

#### Statistical and psychometric analyses

Descriptive statistics were computed to characterize item score distributions. Item response completeness and frequency were examined. Items with high missing values or ceiling/floor effects ( >90% of the ratings in the highest vs. lowest response categories) were excluded from the subsequent analysis since they may reduce the sensitivity and responsiveness of the scale [28].

**Figure 1:**
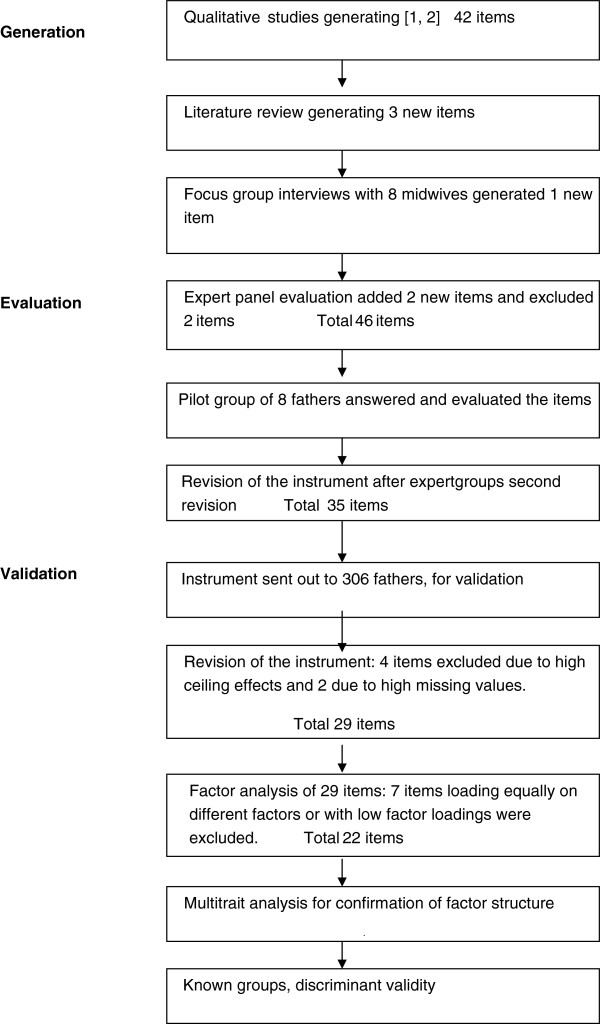
Flow chart of the development and validation of the First-Time Fathers Questionnaire.

A principal components analysis was performed to explore the construct validity of the questionnaire. A varimax rotation was used since the correlations between the components were low (<0.3). Items with maximum loadings less than 0.40 were excluded from subsidiary factor analysis. The Kaiser rule (eigenvalue > 1.0) was applied for determining the number of dimensions to extract, along with the criteria that the factors should be interpretable from a clinical perspective (Figure 1).

Multitrait-scaling analysis was performed to confirm the derived factor structure and to test scaling assumptions for aggregating item ratings of the dimensions. Four assumptions were tested: 1) item internal consistency (item-hypothesized scale correlations ³ > 0.40 and Cronbach’s alpha ³ 0.70), 2) item discriminant validity (item-hypothesized scale correlation > item-other scale), 3) equal item-hypothesized scale correlation (item-scale correlations roughly the same for all items in scale), 4) item equal variance (variances of items in hypothesized scale roughly equal).

Item ratings were aggregated to scale scores for each respondent using the half scale method, i.e., mean values were computed when the respondent had answered at least half of the items in the scale [28].

Known-groups validation [28,29] was used to assess the discriminant validity of the questionnaire, i.e. the ability of the questionnaire to distinguish between subgroups known to differ on key socio demographic or clinical variables. Based on previous research [23,30], it was hypothesized that first-time fathers whose child was delivered with caesarean section would express more negative attitudes than fathers whose child was delivered vaginally. Moreover, it was expected that young fathers [31,32] and immigrant fathers [33,34] would be more distressed, unprepared and in need of more support during childbirth. One-way analysis of variance (ANOVA) was used to compare scale scores between groups and Tukey post hoc tests were used. A 5% significance level was used throughout.

Descriptive statistics (ANOVA) and factor analyses were conducted using SPSS 18 statistical software (SPSS Inc, Chicago, Ill, USA) and the MAP-R program [35] was used for multitrait-scaling analysis.

## Results

### Instrument development

#### Domain identification and item generation

The literature search for domains of fathers’ experiences of childbirth yielded 94 articles, of which 21 were judged to be relevant. Articles were included if they concerned fathers experiences of childbirth and articles in which fathers were not the main focus were excluded. On the other hand, articles about fathers which were related to childbirth but did not focus on labour and birth. Hence, articles that focused on special medical conditions or/and disability of the child were omitted. The literature search yielded no additional domains to supplement the five domains found in our previous interview studies. The focus group interview with the midwives suggested that the identified domains adequately covered fathers’ experiences.

Approximately nine statements representing each domain were extracted verbatim from the interviews. Thus the initial item pool comprised 45 items. The focus group interview added one new item. The evaluation by the expert panel excluded two items, primarily due to overlapping of item content. Based on the panel discussion, two new items were drafted to broaden the coverage of the domains and a number of items were modified to improve item comprehension.

A preliminary version of the questionnaire was drafted comprising 46 items with a 4-point Likert response scale strongly agree (1) agree (2) slightly agree (3) disagree (4). Pilot testing in a group of eight first-time fathers resulted in the deletion of 11 items due to low relevance ratings and in the rewording of several items to improve comprehensibility.

The final questionnaire thus comprised 35 items with an additional 8 questions about how the fathers prepared for birth (2 questions), mode of birth, ethnic background (2 questions), age, level of education and marital status.

### Instrument validation

#### Setting and study sample

**Figure 2:**
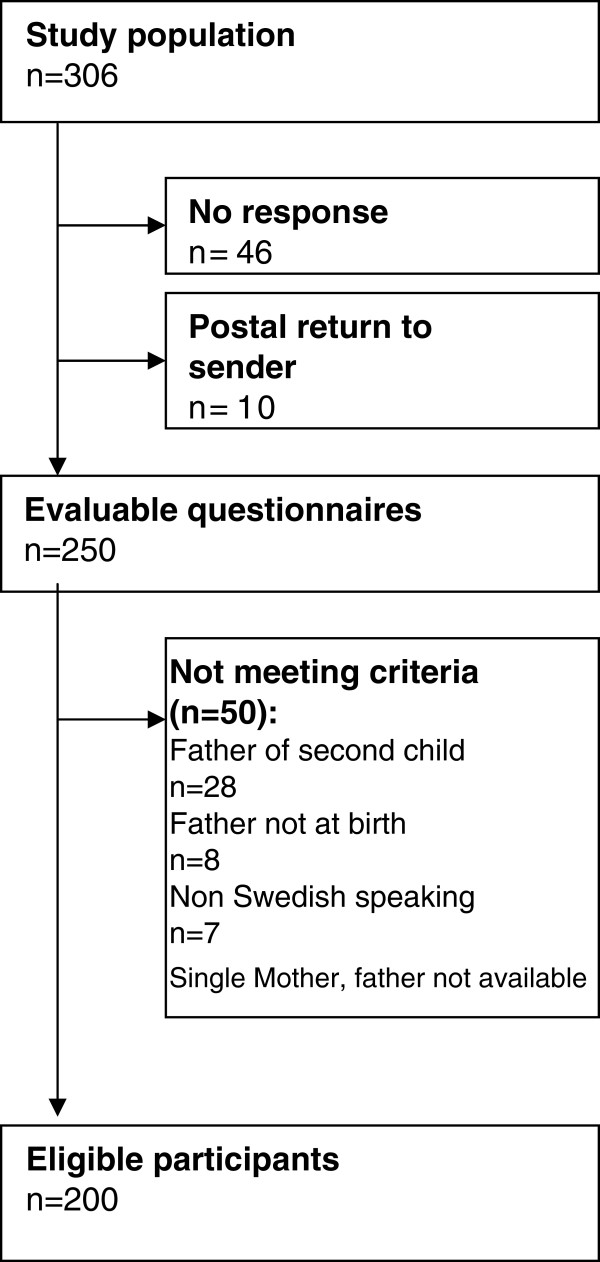
Flowchart of the study sample included in the analyses.

The questionnaire was mailed via the mothers to 306 first-time fathers. Ten letters were undeliverable and 50 fathers did not meet inclusion criteria (Figure 2). Of the remaining 246 fathers, 81% (n = 200) returned the questionnaire after two reminders, 59 of them via the website. The respondents median age was 31 years (Mean 31.8, SD = 5.6) with a range of 19–55 years, which corresponds to the national average of first-time fathers in Sweden [36]. In total, 18% (n = 33) were born outside Sweden (national average 2008 14%), while 24% (n = 46) had one or two parents born outside Sweden (no national average available). A slightly greater proportion of the participants had high school or university education than the national average (Table 1). Nearly all fathers (n = 185) resided with the child’s mother when they answered the questionnaire (no national average available). 

**Table 1:** Socio-demographic characteristics of the study group, n = 200

	**n**	**%**
Marital status (n = 188)*		
Married/ cohabiting	185	97.95
Single	3	1.5
**Educational level (n = 187)**		
Elementary school	15	7.1
High school	81	41
University	91	45
**Mode of delivery 8 (n = 197)**		
Vaginal birth	145	72
Operational vaginal birth	27	13.5
Caesarean Section	25	12.5
Ethnic background (n = 196) **		
Born in Sweden to Swedish parents	155	79
Parents (1 or2) born outside Sweden	46	
Born outside Sweden	33	18
Preparation for Childbirth (n = 198) **		
Antenatal clinic class	158	79
Lamaze class	30	15
Self study	94	47
Consulted friends and family	103	52
No preparation	7	0.5
**Mean age (SD)**	31.8 (5.6)	

· n = number of fathers who answered the question.

** More than one alternative was possible.

#### Item score distributions

Item score distributions were examined for completeness and skewness. Missing value rates were generally low for all items; but one item was excluded from further analysis (missing rate = 92%), i.e.: *‘I had opportunity to touch the baby’s head before it was born’*. Some items had extreme ceiling effects (>90%) and were excluded from further analysis: ‘*I was given adequate information’, ‘I was expected to give support’*, ‘*My role was to hold and massage my partner’, and ‘I was just there, watching the birth’.*

#### Principal components analysis

After excluding skewed items, a total of 29 items were entered in an initial principal components analysis. The analysis yielded nine factors with eigenvalues > 1, which accounted for 62% of the variance (Table 2). However, of these factors five comprised only one or two items, and were judged not to be interpretable from a clinical perspective. Hence a four-factor solution was chosen. In this solution seven items had low or diffuse loadings and were omitted. Examples of these items were: *‘I felt I was in the midwife’s way', 'It didn't make any difference if I was there or not', 'I was mainly a spectator' and 'I was persuaded to participate during childbirth'*. The remaining 22 items yielded 4 factors meeting extraction criteria and explaining 48.6% of the total variance: *Worry, Information, Emotional support, and Acceptance. Worry* (8 items; 20.5% of variance) included items related to concerns about the well being of spouse and infant, inadequacies in giving support, own reactions, and fear of the unknown. *Information* (4 items, 15.6%) comprised items tapping feelings of being prepared and receiving relevant information during childbirth. *Emotional support* (6 items, 6.5%) concerned fathers' experiences of guidance, support and comfort provided by assisting personnel during childbirth. *Acceptance* (4 items, 5.9%) comprised items regarding fathers' impressions of how they were received, treated and acknowledged by health care providers (Table 2).

**Table 2:** Factor analysis

**Items**	**Worry**	**Informed**	**Emotional support**	**Acceptance**
Worry about error	.82			
Worry about the child	.80			
Worry about wife/girlfriend	.72			
Worry about the unknown	.70			
Worry not capable to support	.61			
Worry about own reaction	.58			
I was frighten	.66			
Dispens to take part	.41			
I felt well informed		.78		
I felt well prepared		.74		
Enough information		.66		
Missing some information		.65		
Shown how to hold the baby			.66	
Encouraged to hold the baby			.62	
The HCPs* made sure I was ok			.61	
The HCPs comforted me			.50	
The HCPs gave me guidance in support			.45	
The HCPs replaced me when I needed			.41	
The HCPs received me well				.76
The HCP received me well via telephone				.70
The HCPs gave me positive attention				.47
I felt accepted at the delivery ward				.63

HCPs = Health Care Providers.

#### Multitrait- scaling analysis

The Multitrait-scaling analysis showed that scaling assumptions were adequately met for all dimensions (Table 3). Item-scale correlations exceeded 0.40 for nearly all items and those less than 0.40 were still higher with their own scale than with competing scales (item discriminant validity). Item scale correlations, means and variances showed that items contributed roughly equally to its hypothesized scale. Cronbach’s alpha coefficients were acceptable for group analyses (>0.70) in two scales (*Worry,* 0.82 and *Information* 0.73) and slightly lower in *Emotional support* (0.65) and *Acceptance* (0.66) (Table 3).

**Table 3:** Test of scaling assumptions

**Scale**	**Item internal consistency**				
			**Item**	**Item**	**Item**
	**Item-scale**	**Cronbachs**	**Discriminant**	**Correlations**	**Variance, SD**
	Correlation*	alpha**	**validity**	range***	range ****
Worry	7/8	0.82	8/8	0.34-0.67	0.78-1.17
Information	4/4	0.73	4/4	0.45-0.57	0.74-0.98
Emotional					
support	3/6	0.65	6/6	0.26-0.48	0.92-1.22
Acceptance	4/4	0.66	3/4	0.40-0.49	0.72-0.97

* Item scale correlation > 0.40, corrected for overlap / number of correlations.

** Scale internal consistency reliability (Cronbachs alpha).

*** Item-scale correlations roughly the same for all items in the scale.

**** Item in hypothesized scale have roughly equal variances.

#### Known-groups validation

The discriminant validity of the questionnaire was evaluated by comparing mean scores on each subscale between fathers whose child was delivered with caesarean section (= CS) or instrumental birth (=ID) and those whose child was vaginally born. It is known from earlier research that CS and ID fathers are more worried and less prepared for the situation [23,30]. As expected, the CS and ID group had significantly higher scores (p = 0.000) on the factor *Worry* and the CS had significantly higher scores on the factor *Information* (p = 0.03).

Also as expected [31,37], the youngest fathers had significantly higher scores on the factors *Emotional support* (p = 0.40) and *Acceptance* (p = 0.41) than the oldest fathers. A difference between immigrant fathers versus native Swedish fathers [33,34] was also found, where the immigrant fathers had significantly higher scores on the factor *Worry* (p = 0.013). However, Swedish born fathers with one or two parents born outside Sweden did not significantly differ from those with Swedish born parents. There were no significant differences in any scale due to the father’s educational level (Table 4, 5). 

**Table 4:** Differences in subscale scores between modes of delivery and native Swedes vs. immigrants

**Dimension**	**Vaginal delivery (a) (n = 145)**	**Instrumental delivery (b) (n = 26)**	**Acute Caesarean Section (c) (n = 25)**	**ANOVA, p-value**	**Post hoc a-c**	**Post hoc a-b**	**Native Swedes**	**Immigrants**	P‐value*
Worry	2 21	2.73	2.77	0.000	0 000	0.001	2.29	2.62	0.013
Information	1.72	1.92	2.03	0.032	0.049	0.28	1.76	1.93	0.12
Emotional support	2.36	2.37	2.39	0.96	0.96	0.99	2.38	2.32	0.64
Acceptance	1.37	1.50	1.58	0.18	0.16	0.94	1.41	1.41	0.96

Range: 1‐4, were 1 was the best, and 4 the worst alternative.

**Table 5:** Differences in subscale scores between education and age groups

**Dimension**	**Comprehensive school**	**High school**	**University**	**ANOVA p-value**	**Age 1 <26 n = 31**	**Age 2 26-31 n = 69**	**Age 3 31-37 n = 70**	**Age 4 >37 n = 26**	**ANOVA p-value**	**Post hoc 1-4**
Worry	2.54	2.32	2.30	0.47	2.34	2.32	2.26	2.66	0.10	0.34
Information	1.66	1.81	1.78	0.67	1.85	1.84	1.74	1.66	0.49	0.63
Emotional	2.14	2.48	2.31	0.075	2.65	2.32	2.34	2.22	0.04	0.04
support										
Acceptance	1.45	1.50	1.32	0.083	1.61	1.37	1.42	1.21	0.04	0.02

Range: 1‐4, were 1 was the best, and 4 the worst alternative.

## Discussion

This study reports on the development and validation of an instrument designed to assess first-time fathers' experiences of childbirth. Currently, the only available validated instrument [6] assesses fathers' experiences in a broad perspective, including perceived competence of healthcare providers and the environment in the delivery ward. Moreover, it focuses on fathers in general rather than explicitly on first-time fathers.

Domains and items comprising the instrument were principally derived directly from interviews with first-time fathers, supplemented by literature searches and a focus group interview with experienced midwives. The focus group interview added one item, but it was subsequently excluded in the factor analysis. The literature search was made mainly in medical and nursing databases; however, extending the search to other databases, such as the Sociological Abstracts, might conceivably have added more domains. A draft version of the questionnaire was evaluated by an expert panel and pilot tested in a group of first-time fathers. A revised version was completed by 200 first-time fathers (81% response rate).

Principal components analysis of the questionnaire yielded four domains, reflecting the complexity and multidimensionality of first-time fathers' experiences of childbirth: *Worry, Information, Emotional support and Acceptance* (Table 2). Of the five initial domains identified in our interviews, one could not be corroborated in these analyses, namely *'support to the woman'*. This was due primarily to the fact that many of the items in this domain had extremely high ceiling effects or items hypothesized to belong to this domain were absorbed by the *Emotional support* domain*.*

Most of the variance in the instrument was explained by the domain *Worry*, which confirms the importance attributed to this domain in earlier and more recent research [38,39]. Interestingly, this domain, comprising items about worries and anxiety about the mother and child, corresponds well with the strongest dimension, Discomfort, in [6]. The importance of the second domain, *Information,* has also been confirmed in research [40,41]. Support has often been emphasized in research on the childbirth experiences of fathers in general [13,42,43]; however, our *Emotional support* domain also taps aspects related to guidance and comfort, which has not been accentuated in previous questionnaires. It is noteworthy that the fourth domain, *Acceptance,* accounted for a significant proportion of the variance. Despite the fact that fathers in Sweden have been present during childbirth for decades, the fact that *Acceptance* represented an independent domain suggests that they are still not always well received [18,22,44].

Some items regarding fathers' participation in childbirth and support to their partners had extreme ceiling effects (>90% endorsed the most positive response choice) and were omitted since they were judged to weaken the discriminant validity of the instrument. Possible explanations for the highly positive ratings on these items may be either that father's see their participation and support during childbirth as self-evident or that they wish to present themselves in a positive light and thus respond in a socially desirable manner. Moreover, some of the items about fathers' participation were derived from studies conducted nearly 15 years ago and in a different cultural setting [12,42]. Hence, the items may not be relevant or appropriate for Swedish fathers or for current childbirth practices.

The domains *Emotional support* and *Acceptance* did not meet conventional reliability standards for group comparisons (Cronbach alpha > 0.70). Excluding one or two items in these domains would have yielded acceptable alphas; however, we retained these items because they were considered to be clinically relevant. Multitrait-scaling analysis confirmed that these domains otherwise adequately met scaling assumptions for aggregating item ratings to scale scores and known-groups analyses supported their external validity.

Known groups analyses showed that the instrument could discriminate between subgroups of fathers known to differ on key clinical or socio demographic variables, specifically caesarean vs vaginal birth, older vs younger fathers, Swedish-born vs immigrant fathers, and high vs low education level. Supporting its discriminant validity, significant differences in the expected direction were found in comparisons between these groups, with the exception education level. However, educational level may be a poor indicator of socioeconomic status, which has previously been found to be associated with fathers' experiences of childbirth [3,45,46].

Due to the fact that there is no paternity registry in Sweden, the questionnaire was mailed to the mothers of the newborn with the instruction to give it to the infant's father. This approach may have biased our sample in favour of fathers who currently live with or are on good terms with the mothers. This potential bias may be important since fathers with poor marital relationships have been shown to report higher levels of psychological distress and depressive symptomatology [40,41]. Hence, there may be a need to further validate the questionnaire in a more heterogeneous sample, with respect to marital status.

A strength of the study was that the domains and most items were based on interviews with first-time fathers [15-17], supplemented by a literature search and focus group discussions with experienced midwives. The fact that the items were evaluated iteratively for face validity with regard to comprehensiveness, relevance and comprehension in a panel of experts in paternity research and in a pilot study of first time fathers also supports the validity of the questionnaire. Another strength of the study was the relatively large (n = 296) sample size and the high response rate (81%).

### Conclusions

Our results suggest that this instrument adequately assesses important aspects of first-time fathers' experiences of childbirth. It may serve as a useful and sensitive tool for assessing first-time fathers' experiences and needs at labour wards and may be used to help identify fathers in need of extra support and counselling following negative birth experiences. However, more work is needed to improve the reliability of the instrument, particularly regarding the item content of the domains *Emotional support* and *Acceptance*, before it can be used with confidence for screening purposes.

## Competing interests

The authors declare that they have no competing interests.

## Authors’ contributions

ÅP participated in the design of the study, data collection and analyses, writing the first draft of the manuscript and thereafter in revising and developing the manuscript in collaboration with the co-authors. MB participated in the design of the study and the development of the manuscript and supervision; CT participated in the design of the study, data analyses, supervision and manuscript preparation; and ALH participated in the design of the study and development of manuscript.

## Pre-publication history

The pre-publication history for this paper can be accessed here:

http://www.biomedcentral.com/1471-2393/12/35/prepub
